# Finding a worm's internal compass

**DOI:** 10.7554/eLife.09666

**Published:** 2015-08-05

**Authors:** Catharine H Rankin, Conny H Lin

**Affiliations:** Djavad Mowafaghian Centre for Brain Health and the Department of Psychology, University of British Columbia, Vancouver, Canadacrankin@psych.ubc.ca; Djavad Mowafaghian Centre for Brain Health, University of British Columbia, Vancouver, Canada

**Keywords:** magnetosensation, migration, nematode, *C. elegans*

## Abstract

A pair of neurons is required for nematodes to be able to navigate using the Earth's magnetic field.

**Related research article** Vidal-Gadea A, Ward K, Beron C, Ghorashian N, Gokce S, Russell J, Truong N, Parikh A, Gadea O, Ben-Yakar A, Pierce-Shimomura J. 2015. Magnetosensitive neurons mediate geomagnetic orientation in *Caenorhabditis elegans*. *eLife* **4**:e07493. doi: 10.7554/eLife.07493

**Image** Well-fed worms use the Earth's magnetic field to migrate upwards

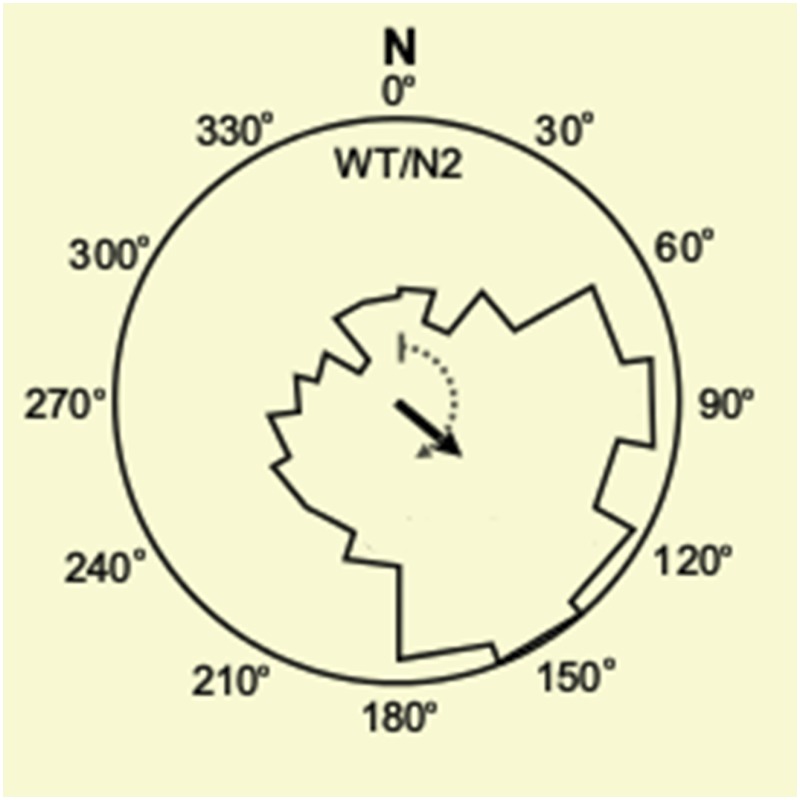

Many animals use internal magnetic compasses to navigate (Johnsen and Lohmann, 2008). Birds and sea turtles use the vertical or horizontal components of the Earth's magnetic field to navigate across large distances (Kishkinev and Chernetsov, 2015), and bacteria use the Earth's magnetic field to migrate vertically (Schüler, 2006). However, controversy remains over where these internal compasses are located within organisms, and how they detect the magnetic field.

Searching for the molecular basis of these internal compasses is not trivial because magnetic fields penetrate virtually all biological tissues (Johnsen and Lohmann, 2008). This means that the compass need not be located on the surface of the body. Many organisms have large structures such as eardrums and lenses to manipulate or focus sound and other cues from the environment. However, since very few biological materials affect magnetic fields, an internal compass is unlikely to have a large structure for this purpose. Thus, the internal compass could be microscopic, contained within cells, and potentially scattered throughout the body.

Now, in eLife, Jonathan Pierce-Shimomura from the University of Texas at Austin and colleagues – including Andrés Vidal-Gadea as first author – use the microscopic worm *Caenorhabiditis elegans* to study magnetic navigation (also known as magnetotaxis). *C. elegans* is a well-established model organism for neurobiology research as it has a simple nervous system with only 302 neurons (White et al., 1986). This makes searching for the neurons that make up the internal compass much simpler than it would be to search through the millions of neurons in birds and the many thousands of neurons in insects.

Vidal-Gadea et al. shielded the worms from external magnetic fields and light, and imposed artificial magnetic fields at controlled angles and strengths. With no magnetic field, the worms migrated randomly. However, when exposed to a magnetic field that was at least half as strong as the Earth's, the worms preferentially migrated in a direction that formed an angle of approximately 132° from the North magnetic pole (Figure 1A). Since this laboratory strain of *C. elegans* was originally isolated in Bristol, UK, this would translate to the worms migrating vertically upwards in their natural environment.

**Figure 1.**
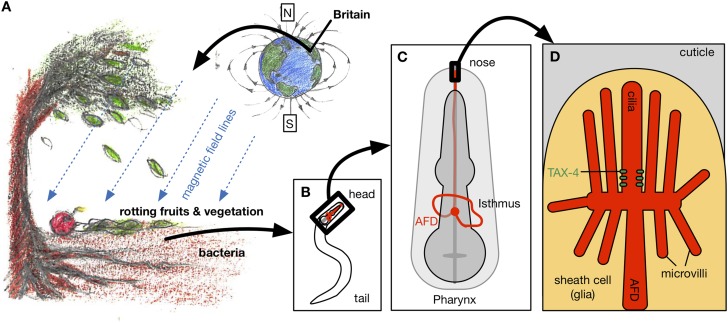
Magnetotaxis in *C. elegans.* (**A**) Worms in the soil migrate upwards at an angle of approximately 132° to the local magnetic field lines (blue lines) in Bristol, UK, perhaps towards the rotting fruits and vegetation on the Earth's surface (Vidal-Gadea et al., 2015). Hungry worms migrate downwards, perhaps towards bacteria near tree roots. (**B**). Magnetotaxis in *C. elegans* requires a pair of neurons called the AFD neurons (shown in red), which are located in the head region of the worm (Altun, 2015). (**C**) AFD neurons wrap around the pharynx (dark grey) and then project towards the nose region. (**D**) The brush-like dendrites at the tip of the AFD neurons are composed of a single cilium and multiple microvilli embedded in a sheath cell just beneath the outer surface of the worm, known as the cuticle (stylized drawing adopted from Perkins et al. (1986); for the actual structure see the electron microscopy reconstruction in Doroquez et al. (2014)). Vidal-Gadea et al. found that an ion channel protein called TAX-4 (green) is required for magnetotaxis. This protein was previously found to be expressed at the base of the AFD cilium (Nguyen et al., 2014).

FIGURE CREDIT: ARTWORK BY CONNY H LIN (CC BY 4.0).

Other strains of *C. elegans* from around the world also migrated at angles that would cause them to move upwards in their original latitude. In addition, the latitude of origin affected the ability of the worms to migrate in response to magnetic fields. Worms from equatorial locations – where the Earth's magnetic fields are weakest – showed poorer magnetotaxis than worms from more northern or southern latitudes. This suggests that there is a lower limit to the magnetic field strength that the worm's compass can detect. Unexpectedly, starved worms shifted their preference by 180°, leading to downward migration, perhaps to find bacteria that they can eat living deeper in the soil.

These data raise an important question: how do worms retain a preference for a particular magnetotaxis direction even after spending many generations at a different latitude? If this preference is due to external or environmental factors that alter the regulation of genes (known as epigenetics), then it would surely be reset to the new location after some generations. Could it be directly encoded by the genetic sequence of the worm? If so, it would suggest that there is a strong adaptive pressure to encode and retain this information.

By examining magnetotaxis in mutant worms that lack responses to particular sensory stimuli, Vidal-Gadea et al. found that a pair of neurons called the AFD neurons – which were already known to carry information about temperature and chemical stimuli from the environment (Mori and Ohshima, 1995) – are critical for magnetic navigation (Figure 1B-C). They used a calcium-sensitive protein to show that the AFD neurons responded to a magnetic field of the same strength as the Earth's, and that another type of sensory neuron did not respond. Studies of worms with mutations in some of the genes expressed in the AFD neurons showed that the *tax-4* gene, which encodes an ion channel protein similar to a photoreceptor found in the retina of human eyes, is required for magnetotaxis (Figure 1D).

In birds, cytochrome molecules in the retina have been proposed to be responsible for detecting magnetic fields. This allows birds to combine light input with magnetic sensors to create a visual-magnetic map for navigation (Kishkinev and Chernetsov, 2015). Since AFD neurons have been implicated in controlling movements made in response to temperature and chemical stimuli (Mori, 1999), this suggests that worms may also combine magnetic field information with other sensory inputs to navigate.

The work of Vidal-Gadea et al. describes an internal compass that resides in an identified neuron in *C. elegans*, and involves an ion channel similar to one involved in vision in humans. This represents a significant advance in our understanding of the neurobiology that underlies how organisms navigate using the Earth's magnetic field. However, it is likely only the beginning of the contributions that studies of *C. elegans* will make to our understanding of how animals navigate using magnetic fields.
